# *In vivo *transcriptional profiling of *Plasmodium falciparum*

**DOI:** 10.1186/1475-2875-3-30

**Published:** 2004-08-05

**Authors:** Johanna P Daily, Karine G Le Roch, Ousmane Sarr, Xuemin Fang, Yingyao Zhou, Omar Ndir, Soulyemane Mboup, Ali Sultan, Elizabeth A Winzeler, Dyann F Wirth

**Affiliations:** 1 Department of Immunology and Infectious Disease, Harvard School of Public Health, Boston, Massachusetts 02115, USA; 2 Department of Cell Biology, The Scripps Research Institute, La Jolla, California 92037, USA; 3 Faculty of Medicine and Pharmacy, Cheikh Anta Diop University, Dakar, Senegal; 4 Genomics Institute of the Novartis Research Foundation, San Diego California, 92121, USA; 5 Department of Department of Biostatistics, Harvard School of Public Health, Boston, Massachusetts 02115, USA

## Abstract

**Background:**

Both host and pathogen factors contribute to disease outcome in *Plasmodium falciparum *infection. The feasibility of studying the *P. falciparum in vivo *transcriptome to understand parasite transcriptional response while it resides in the human host is presented.

**Methods:**

A custom made oligonucleotide array with probes based on the *P. falciparum *3D7 laboratory strain chromosome 2 sequence was used to detect *in vivo P. falciparum *transcripts. This study analyzed transcripts from total RNA derived from small blood samples of *P. falciparum *infected patients and compared the *in vivo *expression profile to the *in vitro *cultivated 3D7 strain transcriptome.

**Results:**

The data demonstrated that *in vivo *transcription can be studied from a small blood sample, despite the abundance of human RNA. The *in vivo *transcriptome is similar to the 3D7 ring stage transcriptome, but there are significant differences in genes encoding a sexual stage antigen and surface proteins.

**Conclusions:**

Whole genome transcription analysis of *P. falciparum *can be carried out successfully and further studies in selected patient cohorts may provide insight into parasite *in vivo *biology and defense against host immunity.

## Background

*Plasmodium falciparum *infection remains a major health problem worldwide. Its complex life cycle has hampered standard methods for the study of pathogenesis. New approaches to elucidate parasite biology using whole genomic methods have provided insight into gene function, transcriptional regulation and stage specific biology [1-4]. Characterization of the *in vivo *biology of this pathogen, through adaptation of a whole genome approach, would provide insight into the host-parasite relationship, parasite virulence factors and inform new strategies for intervention. Genomic scale transcriptional profiling of *P. falciparum *during a natural infection is presented. Small amounts of parasite RNA, isolated from a few milliliters of a blood sample are found to be sufficient for whole genome transcriptional analysis. This data show that several genes are differentially expressed *in vivo*, indicating differences between the transcriptional program of 3D7 laboratory strain parasites growing in culture and naturally occurring infections in the human host.

Whole genome expression has been used in studies of bacterial pathogenesis to identify genes that are specifically transcribed under *in vivo *conditions [5-7]. For example, genes involved in amino acid transport and metabolism are upregulated in *Pasteurella multocida in vivo *as compared to *in vitro *conditions [8]. Similarly, analysis of *P. falciparum *gene expression patterns, particularly the subset of genes that are specifically expressed in the *in vivo *state may identify unique parasite biology when it resides in the host environment. Processes involving parasite metabolism, immune evasion and transmission may be altered in the highly specialized environment of the human host as compared to *in vitro *conditions. In addition, approximately 12% of *P. falciparum's *predicted genes have not been found to be expressed in any of the life cycle stages previously studied [9]. Whole genomic analysis of the parasite *in vivo *may reveal the unique expression of such genes *in vivo*, providing additional targets for intervention.

## Methods

### Parasite isolates

This study was conducted as part of an ongoing *P. falciparum *chloroquine resistance study in Senegal [10]. Patients with mild *P. falciparum *malaria gave consent for the study and were enrolled at an outpatient health clinic. Patients underwent venipuncture using K_3 _EDTA coated Vacutainers (Beckton Dickinson) and from this sample, 1.6–2.5 ml of whole blood was collected and passed through a white cell depletion filter using a 20 ml syringe. The filtered sample was centrifuged for 5 minutes at 3,200 rpm in a clinical centrifuge and placed in Tri-Reagent BD (Molecular Research Center). The samples were vortexed and stored at minus 70°C. Samples were thawed in a room temperature bath one month later and isolation of RNA was completed per Molecular Research Centers protocol. Three samples obtained in Senegal that had the highest parasitemia and largest blood volume are presented. A 14 ml blood sample from a *P. falciparum *infected traveler from Nigeria was similarly processed in Boston.

### Oligonucleotide array analysis

Labeling and hybridization of total RNA was performed as described [2]. Expression levels were calculated using the Match Only Integral Distribution Algorithm (MOID) [11]. The presence or absence of gene expression was determined using methods previously described [9]. The design of the probes to human ESTs was based on UniGene version 116.

### Real time PCR

To confirm array data, a subset of genes (PFB0120, PFB0100, PFB0270 PFB0355, and PFB0065) that vary from high to low level abundance by array were quantified using real time PCR from cDNA. PFB0120: forward primer 5'-CAG CCC TCT TAG CTC TCA ACT TC-3', reverse primer 5'-AGC AAC AGC AGA GGC TAT AGA ACT-3', PFB0100: forward primer 5'-CAC CAA ATG GCT ATG CTT ATG GA-3', reverse primer 5'-TTC CAG GAG CAC CAT TAA ATC CT-3', PFB0270: forward primer 5'-ACA CTT ACT GGT ATT TCG GAA TTT-3', reverse primer 5'-TAA TTG TCC ATA TTC TTC AAT ATA T-3', PFB0355: forward primer 5'-ATT GTA AGA AAT AGT TGG GGT-3', reverse primer 5'-TAT ATC ATG CTC CTT CTT ATC A-3', PFB0065: forward primer 5'-CGT TGG TAG TGC GTT CCT TAC AA-3', reverse primer 5'-GTT CCT GCT ATA TCA GGA GCA CCA-3'. Sequence analysis confirmed the identity of the amplification products. 3D7 strain parasites were cultivated under standard conditions and synchronized with 5% sorbitol to obtain ring stage parasites for extraction of total RNA [12,13]. cDNA was synthesized from total RNA from the Nigerian *in vivo *sample and 3D7 ring stage total RNA using Super Script 1^st ^Strand synthesis system (Invitrogen). Duplicate reactions using real time PCR were performed with 1 μl cDNA with gene specific primers in 50 μl reaction volume using fluorescent dye SYBR Green (SYBR Green PCR Master Mix, Applied Biosystems). The reactions were carried out on an ABI PRISM model 7700-sequence detector and all PCR reactions amplified a single product as determined by dissociation curve analysis (Dissociation Curve Software, Applied Biosystems).

### Statistical tests

Variation between samples was assessed using Kruskal-Wallis method (non-parametric ANOVA) to test the null hypothesis. To normalize samples, the mean gene expression level was calculated for all *Plasmodium *genes between the 10^th ^and 90^th ^percentile with at least six probes. Analysis based on rank was a second method used; in each experiment the probe intensity was ranked and this resulted in equivalent quantile distribution for all probes between two experiments. This method is more conservative and will define relative rank changes between experiments and is independent of potential normalization artifacts.

### Human subjects

Patient blood samples were collected after informed consent was obtained. The study was approved by the institutional review boards at Harvard School of Public Health, Brigham and Women's Hospital and Cheikh Anta Diop University.

## Results

**Figure 1:**
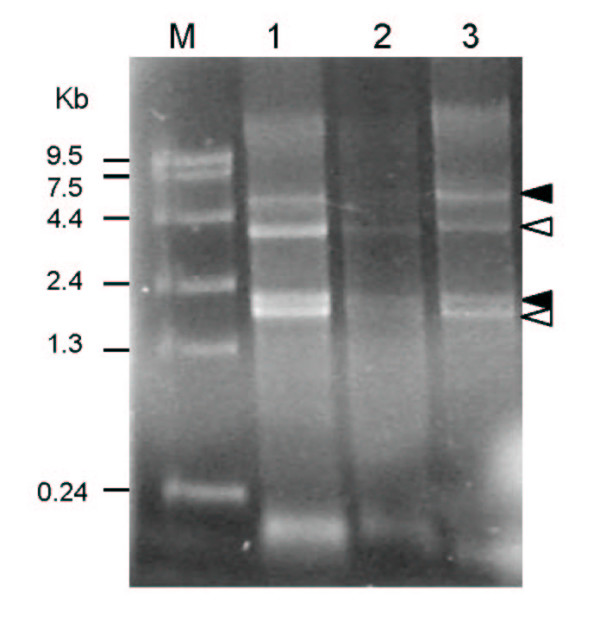
Denaturing gel of total RNA isolated from *P. falciparum *infected patient blood reveals both human and parasite ribosomal RNA. Total RNA was electrophoresed on a 1.3% formaldehyde agarose gel and stained with ethidium bromide. Marker (M) marks RNA size ladder. Three patient samples were run in lane 1–3. Closed arrows represent human ribosomal RNA 28s: 4700 bp, 18s:1900 bp; Open arrows mark *P. falciparum *ribosomal RNA 28s:4104 bp, 18s:1384 bp.

To evaluate the integrity of the RNA transcripts from the *in vivo *isolated samples a denaturing RNA gel was carried out (Figure 1). The ribosomal bands are sharp with minimal RNA degradation. Despite buffy coat depletion there are human ribosomal bands present in addition to *P. falciparum *ribosomal bands. Human ribosomal bands are not seen on a denaturing RNA gel from *in vitro *cultivated 3D7 (data not shown). The most abundant transcript of human origin in the *in vivo *was haemoglobin RNA (Table 1). Human transcripts are also detected in 3D7 *in vitro *samples, but at a lower level of abundance.

**Table 1:** Human transcripts detected in *P. falciparum *infected patient blood samples and *in vitro *cultivated 3D7 samples Comparison of expression level of the most abundant human transcripts detected in *in vivo *blood and in 3D7 *in vitro *cultivated samples. Average expression level of *in vivo *isolated RNA derived from the average expression level of three samples from Senegal and one from Nigeria compared with the average expression level from three 3D7 ring stage *in vitro *isolated RNA samples. Transcripts are listed in order of highest abundance *in vivo *as detected by array. Expression levels are reported as expression units (EU), using the MOID algorithm.

		**Expression Units**	
		
**Gene**	**Description**	***in vivo***	***in vitro *3D7**
Hs.155376_at	haemoglobin, beta	757266	101307
Hs.36977_at	haemoglobin, delta EST, similar to tctp human translationally controlled tumor protein *H. sapiens*	395849	12265
Hs.203820_at		241506	6932
Hs.21295_at	EST, Weakly similar to KIAA0902 protein *H. sapiens*	190454	9321
Hs.247921_at	haemoglobin, theta 1	188961	4528
Hs.87246_at	Bcl-2 binding component 3	187575	5688
Hs.256047_at	ESTs, similar to B24264 proline-rich protein MP3 -*M. musculus*	160290	5681
Hs.14587_at	ESTs, similar to AF1 51 859_1 CGI-101 protein *H. sapiens*	155768	6389
Hs.117848_at	haemoglobin, epsilon 1	150921	5903
Hs.168073_at	DKFZP727M231 protein	147492	6615
Hs.24545_at	hypothetical protein FLJ11137	146324	6292
Hs.6318_at	peroxisomal short-chain alcohol dehydrogenase	145846	5136
Hs.155833_at	ESTs, similar to spliceosomal protein SAP 155 *H. sapiens *Homo sapiens mRNA; cDNA DKFZp434H0820 (from clone DKFZp434H0820); partial cds	140563	5508
Hs.109857_at		138856	7397
Hs.248677_at	ESTs, similar to A48018 mucin 7 precursor, salivary – *H. sapiens*	136390	5253
Hs.4205_at	hypothetical protein FLJ20124 ATPase, H+ transporting, lysosomal (vacuolar proton pump), member D	132244	4050
Hs.106876_at		127582	3930
Hs.172914_at	retinol dehydrogenase 5 (11-cisand 9-cis)	125310	4560
Hs.23898 at	paraneoplastic antigen	124648	5631

**Figure 2:**
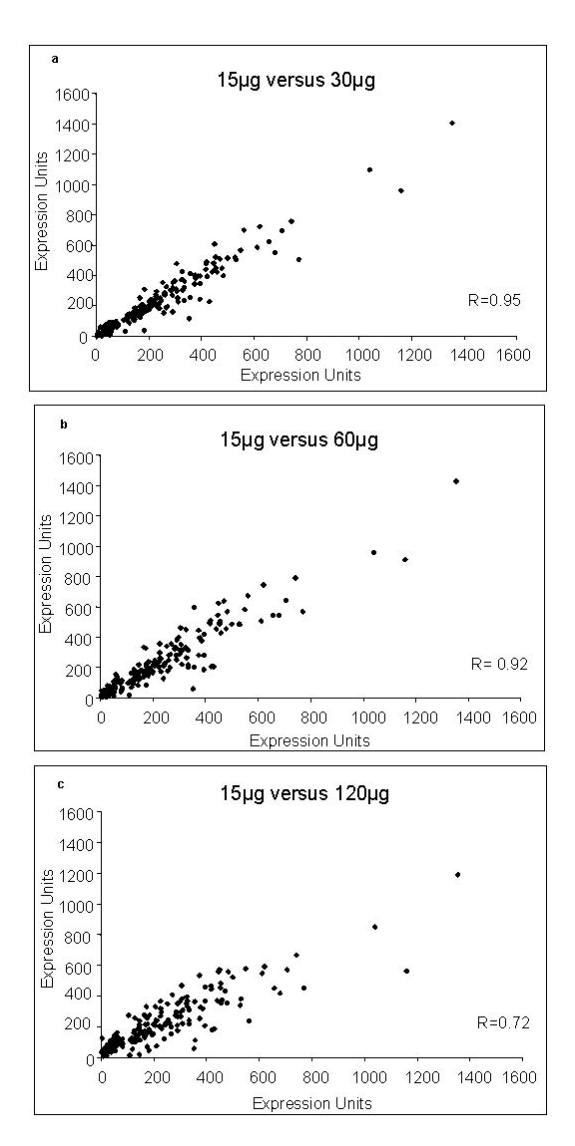
Scatter plot of expression level variance between samples to define the highest signal to background ratio of calculated expression units for *P. falciparum *probes. Increasing concentrations of cRNA from the Nigerian sample were hybridized to the array and expression levels (Expression Units) for each transcript were derived using the MOID algorithm. 15 μg cRNA data is presented on the Y axis (**a**) 15 μg v. 30 μg of starting cRNA. (**b**) 15 μg v. 60 μg starting cRNA. (**c**) and 15 μg v. 120 μg starting cRNA. (R = correlation coefficient).

The corresponding peripheral blood smears for the four *in vivo *samples contained only ring forms. Notably, only ring stages are present in the peripheral blood of *P. falciparum *infected patients; later stages are sequestered in the microvasculature. For this reason, the *in vivo *whole genome transcription data was compared to the *in vitro *chromosome 2 ring stage transcriptome. Three samples with parasitemias that were less than 0.3% and total volumes of up to 2.5 ml from Senegal were studied: this resulted in the detection of fewer transcripts than the sample obtained from a Nigerian patient who had parasitemia of 0.4% and underwent a larger blood draw. However, 50% of the top twenty five expressed transcripts in all four samples were shared (data not shown). Further analysis was performed on the Nigerian sample. Only one parasite line was detected in this sample through DNA genotyping of the K1, MAD20, RO33 alleles of *msp1 *and FC27 and IC1 alleles of *msp2 *using primers and methods previously reported [14]. After total RNA was isolated, aliquots of 8 μg of total RNA were labeled using a modified Eberwine procedure [2]. To maximize parasite transcript detection, 15 μg to 120 μg of cRNA were hybridized to the array and a quantitative expression level was calculated using the MOID algorithm for the *Plasmodium *genes on the array [2]. Correlation coefficients comparing *P. falciparum *chromosome 2 expression levels utilizing 15 μg to 30 μg or 15 μg to 60 μg cRNA were 0.95 and 0.92, respectively. However, as cRNA concentration was increased to 120 μg, the background to noise ratio increased significantly, resulting in a decreased correlation coefficient (R = 0.72) (Figure 2a,2b and 2c).

The most abundant transcripts detected from the Nigerian *in vivo *sample are listed in Table 2. Genes in bold are uniquely expressed in the *in vivo *sample compared to 3D7 ring stage previously reported using the Kruskal-Wallis method [2]. Notably, a number of genes encoding surface proteins such as rifins and SERA antigens appear overexpressed *in vivo*.

**Table 2:** *P. falciparum *genes expressed *in vivo *encoded by chromosome 2. *In vivo *transcripts from the Nigerian sample were defined as present as compared to uninfected blood control hybridization. Asterisk (*) denotes transcripts that were also detected in a Senegal derived blood sample. Genes in bold are uniquely expressed *in vivo *and were not found to be expressed in the previously reported 3D7 ring stage transcriptome. Gene locus is from PlasmoDB 4.1 .

gene locus	description	gene locus	description
**membrane proteins**		**cellular function**	
PFB0120w*	etramps 2	PFB0175c*	prt of the MAK16 family
PFB0405w*	**transmission blocking target antigen**	**PFB0205c**	**prt with 5'-3' exonucl. domain**
**PFB0015c**	**rifin**	PFB0210c	monosaccharide transporter
**PFB0025c**	**rifin**	PFB0265c*	RAD2 endonucl.
**PFB0035c**	**rifin**	PFB0295w*	adenylosuccinate lyase (OO)
PFB1010w*	**rifin**	PFB0380c	phosphatase (acid phosphatase family)
PFB1020w*	**rifin**	PFB0390w*	**ribosome releasing factor (OO, TP)**
PFB1035w*	**rifin**	PFB0410c*	**phospholipase A2-like a/b fold hydrolase**
PFB1050w*	**rifin**	PFB0445c	elF-4A-like DEAD family RNA helicase
**PFB0330c**	**SERA antigen/papain-like protease**	**PFB0510w**	**GAF domain prt**
PFB0340c	SERA antigen/papain-like protease	PFB0525w	asparaginyl-tRNA synthetase (OO, TP)
PFB0345c*	SERA antigen/papain-like protease	**PFB0585w**	**Leu/Phe-tRNA prt transferase**
PFB0355c*	**SERA antigen/papain-like protease**	PFB0595w*	prt with DnaJ domain, DNJ1/SIS1 family
**PFB0360c**	**SERA antigen/papain-like protease**	PFB0760w	Mtn3/RAG1IP-like prt
**PFB0095c**	**membrane protein, PfEMP3**	**PFB0795w**	**ATP synthase alpha chain**
**PFB0975c**	**PfEMP1 fragment**	PFB0815w*	calcium-dept. prt kinase
PFB1045w	Pf EMP1 fragment	PFB0875c	chromatin-binding prt (SKI/SNW family)
PFB1055c	PfEMP1 (var gene)	**hypothetical proteins**	
PFB0085c*	prt with DnaJ domain (RESA-like)	**PFB0135c**	**hypothetical protein**
PFB0100c*	knob-associated His-rich prt	PFB0490c*	hypothetical protein
PFB0300c	merozoite surface antigen MSP-2	PFB0575c	hypothetical protein
**PFB0475c**	**predicted multiple-TM membrane prt**	**PFB0580w**	**hypothetical protein**
PFB0770c*	**predicted multiple-TM membrane prt**	PFB0630c	hypothetical protein
**PFB0125c**	**predicted membrane associated prt**	PFB0705w	hypothetical protein
PFB0735c	predicted integral membrane protein	PFB0745w	hypothetical protein
PFB0275w*	membrane transporter	PFB0870w	hypothetical protein
**PFB0465c**	**membrane transporter**	**other**	
		PFB0675w	predicted secreted protein
		PFB0990c	predicted secreted protein

**Figure 3:**
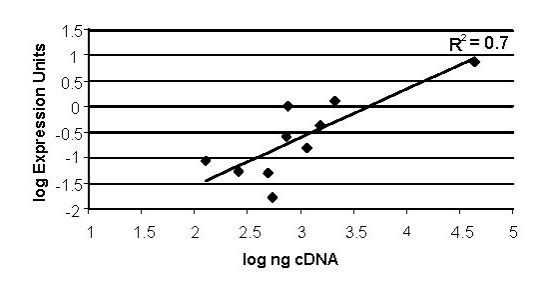
Correlation of expression levels derived from the oligonucleotide array with ng cDNA determined by real time PCR. Log ng of cDNA from the Nigerian sample and the *in vitro *3D7 ring stage sample was determined for five genes that vary from high to low level abundance by array: PFB0120, PFB0100, PFB0270, PFB0355, and PFB0065. cDNA concentration was determined with real time PCR using a standard curve based on 3D7 genomic DNA. These results were correlated to the expression units (EU) found by the array. (R^2 ^= correlation coefficient)

To confirm the accuracy of the results from the oligonucleotide array and to confirm the *in vivo *overexpression of a SERA antigen (PFB0355), the relative expression of five genes that had varying transcript abundance by array was carried out using real time PCR of cDNA generated from total RNA isolated from the Nigerian *in vivo *sample and a 3D7 *in vitro *ring stage sample. There is good correlation between the array results and those obtained by real time PCR (Figure 3). To compare abundance of PFB0355 cDNA between the *in vivo *and *in vitro *samples, the data is normalized to cDNA of PFB0120 to account for differences in starting parasite cDNA, secondary to human cDNA. The *in vivo *sample contained 0.15 ng cDNA of PFB0355c and 3D7 ring stage cDNA had 0.09 ng by real time PCR. When PFB0355c is normalized to PFB0120c, it was found to be ten fold overexpressed *in vivo *as compared to *in vitro*, consistent with the array results.

## Discussion

This data confirms that *in vivo *whole genomic expression can be performed despite the potential technical challenges of scarce RNA contained in a small blood volume sample and presence of abundant human RNA. Tri-Reagent BD was used to stabilize RNA and the samples were stored at -80°C before transport to the US. The denaturing gel suggests that the RNA remains intact using this reagent. Notably Kyes et al. reported that minus 80°C rather than 4°C or minus 20°C is the optimal temperature to store field sample RNA for detection of long transcripts such as *var *[15]. Surprisingly, abundant human RNA was detected in the denaturing gel despite buffy coat depletion of the samples. In addition, the degree of hybridization to human probes on the oligonucleotide array used here was not seen in the previously studied *in vitro *sample [2]. Haemoglobin is found to be the most abundantly expressed human transcript (Table 1) with considerably higher levels noted in the *in vivo *samples as compared to the 3D7 *in vitro *sample. Although human red cells are used for culturing in the *in vitro *system, reticulocytes which contribute to the haemoglobin detected are not abundant in *in vitro *samples. This is most likely due to the observation that reticulocyte levels display a 75% loss at 48 hours, when placed at 37°C, which is the condition of *in vitro *culture [16]. In addition, other human RNA such as ribosomal RNA is more abundant in the *in vivo *samples and may be secondary to white cell contamination. Cross hybridization of human RNA to parasite probes may occur. However, Zhou *et al *have shown that human genomic DNA does not highly cross hybridize to the parasite probes on this custom array [17]. The high specificity of this array is due to the nature of the highly AT rich parasite genome as compared to the human genome and the careful selection of parasite unique 25 mers probes [2,18,19]. Due to this high specificity, it is likely that buffy coat depletion is not necessary for analysis of *in vivo *parasite transcripts when using these probes.

The amount of blood volume necessary for comprehensive detection of transcription depends on level of parasitemia and method of microarray analysis. This analysis utilized the Affymetrix system which requires very little starting RNA as compared to other methods [20]. Due to the presence of human RNA in the *in vivo *samples it was not possible to determine how much parasite RNA is required for whole genome analysis. The samples from Senegal were of low parasitemia and small volumes, whereas the 14 ml blood sample with 0.4% parasitemia was sufficient to detect a greater number of chromosome 2 transcripts. Four to five mls of packed blood in a patient with a greater than 2% parasitemia should provide sufficient material for whole genome analysis using these methods. The data demonstrates the reproducibility of the method by independent hybridizations and that maximal senstivity can be achieved with up to 60 μg of cRNA, using this array.

PFB0120w is the most abundant *in vivo *transcript in all samples encoded on chromosome 2. This gene is a member of a recently described gene family, *etramps*, expressed at early ring stage encoding a protein thought to be involved in erythrocyte remodeling; this was also the most highly expressed transcript in *in vitro *ring stage cultures [2,21,22]. The *in vivo *expressed genes from the Nigerian sample was compared to that of the *in vitro *3D7 ring stage chromosome 2 transcriptome [2]. As expected, there was a good correlation between the *in vivo *ring stage transcriptome and the 3D7 ring stage transcriptome. Most of the genes that are expressed *in vivo *are also expressed *in vitro *particularly those involved in cellular function and genes encoding hypothetical proteins (Table 2) [2]. A number of differentially expressed genes involved in transmission and antigenic variation were identified. There is high transcription level of transmission blocking target antigen (PFB0405w) in the *in vivo *samples. Previously this gene has been demonstrated to be expressed and transcribed only in the sexual transmission stage [3,9]. Since no transmissible forms were identified by microscopy this suggests that the *in vivo *samples may have more parasites that are undergoing or are committed to sexual development than is detectable by microscopy. Several genes encoding membrane proteins, including *SERA *and *rifin *genes, were also found to be differentially expressed. These genes are members of multigene family that encode surface proteins which are thought to be involved in immune evasion [23,24]. The observed increase in transcription for these genes *in vivo *could be due either to genetic or transcriptional modulation of the parasite's defense repertoire or geographic variation. Differences between the *in vivo *and *in vitro *expression of SERA antigens may be due to differences in the *in vivo *stage of development as this gene family is transcribed at later stages in the *in vitro *life cycle [2,4,25]. There was overall higher hybridization intensity of the *in vitro *samples due to higher parasite counts and a subset of genes were found to be overexpressed *in vitro *after normalization. This analysis focussed on genes overexpressed *in vivo *as these results would not be influenced by normalization algorithms. Overall, this data demonstrates that *P. falciparum *transcripts can be detected from *in vivo *samples, and that there are potentially important differences between trancription of *in vivo *samples and that of the 3D7 *in vitro *trancription profile.

In summary, this study provides evidence that whole genome gene expression in *P. falciparum *can be studied *in vivo *from a small blood sample of an infected patient. The *in vivo *sample however contains human RNA, whose quantity may vary from sample to sample and therefore differenes in parasite transcript level between samples must be reported relative to a reference transcript. Despite the abundance of human RNA the genomes are sufficiently different with resultant probes specificity. Predictably, there was a high correlation of *in vivo *expression with the *in vitro *ring stage 3D7 transcriptome [2]. Importantly these data also suggest differences between *in vivo *and *in vitro *expression levels in genes typically found in transmissable forms and encoding variant surface proteins. Evaluation of trancription of genes specific for gametocyte development in specific patient populations may uncover the *in vivo *conditions that favor development of transmissable forms. Similarily, a whole genome analysis can comprehensively characterize expression of multigene families that encode variant surface proteins under *in vivo *conditions. Further exploration of the *in vivo *biology of *P. falciparum *using specific probes to all annotated genes will be undertaken to confirm and explore other important biological differences. This new approach will further the understanding of the host-pathogen interaction and may result in the development of new strategies to combat this disease.
